# Differential MicroRNA expression following head‐down tilt bed rest: implications for cardiovascular responses to microgravity

**DOI:** 10.14814/phy2.14061

**Published:** 2019-05-13

**Authors:** Carl J. Ade, Debra A. Bemben

**Affiliations:** ^1^ Department of Kinesiology Kansas State University Manhattan Kansas; ^2^ Department of Health and Exercise Science University of Oklahoma Norman Oklahoma

**Keywords:** Bed rest, cardiovascular, circulating microRNA, c‐miRNA, microgravity, MicroRNA

## Abstract

Head‐down tilt bedrest (HDBR), an analog of spaceflight, elicits changes in cardiovascular function that adversely affect astronaut performance. It is therefore fundamental to elucidate the molecular regulators of these changes. Study aim was to determine if cardiovascular‐related circulating microRNA (miRNA) are altered following HDBR and if they relate to changes in cardiac function and peak aerobic capacity. Eleven participants completed 30‐days HDBR at an ambient CO_2_ of 0.5% (replicate the in‐flight CO_2_ levels). Blood samples were obtained 3 days (BDC‐3) prior to and immediately (R + 0) following HDBR. 44‐targeted circulating miRNAs (c‐miRNA) identified from published roles in cardiovascular structure/function were analyzed via RT‐qPCR. Resting stroke volume was evaluated via ultrasonography. Peak oxygen uptake ($\dot{V}O_{2}\text{peak}$) was determined using a graded exercise test on an electronically braked cycle ergometer. Ten cardiovascular‐related miRNA were significantly increased following HDBR. The differentially expressed c‐miRNA were grouped into clusters according to their expression profile. Cluster A included c‐miRNA that have been identified as regulators of cardiac function and hypertrophy (c‐miRNA‐133), atrial fibrillation and mitochondrial function (c‐miRNA‐1), skeletal muscle atrophy (c‐miRNA‐1), and vascular control (c‐miRNA‐155). Cluster B contained c‐miRNA identified as regulators of cardiac hypertrophy (c‐miRNA‐30, ‐15), fibrosis (c‐miRNA‐22, ‐18), mitochondrial function (miRNA‐181), and aerobic capacity (c‐miRNA‐20a). Following HDBR resting stroke volume was decreased and correlated with changes in c‐miRNA‐378a and ‐18a. $\dot{V}O_{2}\text{peak}$ was decreased and correlated with changes c‐miRNA‐133. In conclusion, we found that HDBR induced a distinct and specific cardiovascular‐related miRNA response, which were associated with changes in cardiac function and peak aerobic capacity.

## Introduction

Prolonged removal of gravitational forces with actual or simulated weightlessness (e.g., head‐down‐tilt bed rest, HDBR) has the potential to elicit numerous molecular and systems level changes that may negatively influence short‐term mission success and long‐term astronaut health. Given the significant changes in systems level cardiovascular function associated with microgravity exposure and the recent molecular changes observed as part of the 1‐year TWINS study (Mason 2018; Mishra et al. 2018; Rizzardi et al. 2018), a continued need to increase our understanding of the molecular regulators of these responses and their clinical utility for future long‐duration exploration missions is required. As such, it is of paramount importance to quantify the changes in the cellular/molecular, structural, and functional properties following microgravity exposure.

MicroRNA (miRNA) represent a class of short (~21–22 nucleotides in length) noncoding RNA that regulate gene expression post‐transcriptionally by targeting messenger RNA at the 3’‐untranslated region and elicit translational repression or degradation (Ambros 2004). Many miRNAs are linked to specific biological functions, originate from specific tissues, and often have an essential role in mediating intracellular processes (Laterza et al. 2009; Fichtlscherer et al. 2010; Madrigal‐Matute et al. 2013), particularly within the cardiovascular and musculoskeletal systems. Importantly, certain miRNA are capable of entering the bloodstream via several mechanisms in response to various stressors including tissue injury and adaptation (Madrigal‐Matute et al. 2013) and provide insight into the molecular signaling associated with physiological outcomes. These “circulating” miRNA (c‐miRNA) have great potential as a biomarker of physiologic adaptation as several c‐miRNA are tissue‐specific, have a long half‐life, are easily detectable, and require minimally invasive procedures (i.e., venous blood sample) (Madrigal‐Matute et al. 2013).

To date, distinctive c‐miRNA profiles have been identified as critical mediators to cell stress and diseases such as myocardial infarction, heart failure, atherosclerosis, coronary artery disease, and a variety of other cardiovascular disorders (Jiang et al. 2009; Mendell and Olson 2012). For example, several investigations have demonstrated increases in c‐miRNA‐208, c‐miRNA‐499, c‐miRNA‐1, and c‐miRNA‐133 in plasma samples of patients in the initial hours following acute myocardial infarction and left ventricular injury (Bostjancic et al. 2010; Corsten et al. 2010; D'Alessandra et al. 2010; Wang et al. 2010; Kuwabara et al. 2011). Similarly, c‐miRNA has been shown to be strongly related to the clinical diagnosis of heart failure (Tijsen et al. 2010). Cardiac plasticity is also mediated by several miRNA, many of which are known to enter the blood stream (i.e., c‐miRNA18b and c‐miRNA‐23a) (Tatsuguchi et al. 2007). Similar to disease‐related outcomes, unique c‐miRNA profiles have been observed with nonpathological adaptation. Baggish et al. (2011) demonstrated a significant increase in c‐miRNAs associated with antiangiogenesis following 90 days of exercise training in 10 student athletes (Baggish et al. 2011), with several c‐miRNA significantly correlated with aerobic exercise capacity, which is known to decrease with prolonged bedrest (Ade et al. 2015). Additionally, Mooren et al. (2014) demonstrated that the c‐miRNA‐133a, a suggested regulator of cardiac hypertrophy (Care et al. 2007), was significantly correlated with intraventricular septum thickness (Mooren et al. 2014) and aerobic exercise capacity. These findings highlight the potential effects of miRNAs on the well‐established cardiovascular adaptations associated with prolonged bed rest. Given these previous findings quantification of cardiac‐specific c‐miRNAs may allow for manifestations of asymptomatic cardiovascular changes to be identified very early and be used to predict cardiovascular‐related outcomes, specifically changes in left ventricular function and aerobic exercise capacity. Therefore, the primary aim of this study was to determine if cardiovascular‐health related c‐miRNA are altered following 30 days HDBR and to determine how they relate to changes in cardiac function and maximal aerobic capacity.

## Methods

### Participants

Eleven participants (five women/six men) completed a 30 day HDBR period at the :envihab bed rest facility located at the institute for Aerospace Medicine in Cologne Germany. Participants had a mean age of 33 years (range 25–50 years), a mean height of 174 cm (range 158–186 cm), and a mean weight before HDBR of 71 ± 9 kg and a mean weight after HDBR of 69 ± 8 kg (*P* = 0.0001). General body composition was evaluated via dual‐energy X‐ray absorptiometry was performed 3 days prior to [baseline data collection (BDC‐3)] and immediately upon completion of the bed rest period [recovery day 0 (R + 0)]. All participants were free from known cardiovascular, pulmonary, or metabolic disease and were nonsmokers as determined from a health history questionnaire. All subjects provided written informed consent to participate in the study. The study was approved by the NASA and Kansas State University Institutional Review Boards for Research Involving Human Subjects.

### Study design

The bed rest consisted of three phases: (1) baseline control experiments pre bed rest; (2) a 30 day period of −6° head‐down tilt bed rest without countermeasures with an ambient CO_2_ of 0.5% consistent with the average levels on the International Space Station (Law et al. 2014); (3) post bed rest experiments. Blood sampling for complete blood count and c‐miRNA analysis was performed on BDC‐3 and immediately upon completion of the bed rest period (R + 0). All blood samples were obtained following an overnight fast with no exercise 8 h prior. Resting cardiac variables were obtained on BDC‐5 and R + 0. Peak oxygen uptake testing was performed on BDC‐4 and R + 0. General body composition was evaluated via dual‐energy X‐ray absorptiometry on BDC‐3 and R + 0. Throughout the HDBR study period participants maintained a strict −6° head‐down tilt position, which was verified 24/7 for the duration of the bed rest phase. Dietary intake was a balanced intake of macro and micronutrients that was planned and supervised by a registered dietitian.

### Circulating miRNAs

All blood miRNA analyses were conducted at TAmiRNANA (Vienna, Austria). Total RNA was extracted from serum/plasma samples using the miRNANeasy Mini Kit (Qiagen, Germany). For each sample, 200 *μ*L of serum were mixed with 1000 *μ*L Qiazol and 1 *μ*L of a mix of 3 synthetic spike‐in controls (Exiqon, Denmark). After a 10‐min incubation at room temperature, 200 *μ*L chloroform were added to the lysates followed by cooled centrifugation at 12,000*g* for 15 min at 4°C. Precisely 650 *μ*L of the upper aqueous phase were mixed with 7 *μ*L glycogen (50 mg mL^−1^) to enhance precipitation. Samples were transferred to a miRNANeasy mini column, and RNA was precipitated with 750 *μ*L ethanol followed by automated washing with RPE and RWT buffer in a QiaCube liquid handling robot. Finally, total RNA was eluted in 30 *μ*L nuclease‐free water and stored at −80°C until further analysis. RT‐qPCR was used to quantify 44 targeted miRNANAs identified from published roles in cardiovascular structure/function. Briefly, starting from total RNA samples, cDNA was synthesized using the Universal cDNA Synthesis Kit II (Exiqon, Denmark) according to manufacturer recommendations. PCR amplification was performed in a 384‐well plate format using custom Pick & Mix plates (Qiagen, Germany) in a Roche LC480 II instrument (Roche, Germany) and EXiLENT SYBR Green mastermix (Exiqon, Denmark) with the following settings: 95°C for 10 min, 45 cycles of 95°C for 10 sec and 60°C for 60 sec, followed by melting curve analysis. To calculate the cycle of quantification values (Cq‐values), the second derivative method was used. Cq‐values were normalized to the mean Cq‐value in each sample (global mean normalization) by subtracting the individual miRNA Cq‐value from the Cq average calculated for that sample. Hemolysis was assessed in all samples using the ratio of miRNA‐23a‐3p versus miRNA‐451a and applying a cut‐off of > 7 to the ratio for calling a sample hemolytic (Blondal et al. 2013).

Functional enrichment analysis was performed on the miRNA that were differentially expressed in response to bed rest in order to further understand their biological relevance to cardiovascular physiology and pathology. This analysis was performed in the context of the enriched KEGG and REACTOME pathways and Gene Ontology (GO) terms based on experimentally confirmed miRNA targets using the miRNANet network‐based visual analysis web‐based tool (Fan et al. 2016). A *P*‐value cut‐off of < 0.05 was used to identify enriched processes associated with cardiovascular physiology.

### Complete blood count

Whole blood was collected from the Antecubital vein into EDTA sample collection tubes. Complete blood counts were then analyzed using standard procedures at the :envihab facility.

### Cardiac function

Resting stroke volume was evaluated via 2D Doppler ultrasonography of the ascending aorta. Briefly, the a pulse wave Doppler ultrasound transducer (1.9 MHz) was positioned at the suprasternal notch and used to obtain measurements of ascending aortic blood velocity time integral. Two dimensional images of the aortic annulus diameter were obtained from the parasternal long axis. All images were stored digitally and used to calculate stroke volume as annulus diameter × velocity time integral. Heart rate measures are derived from the simultaneous ECG during Doppler imaging. Resting cardiac output was calculated as stroke volume × heart rate.

### Peak oxygen uptake ($\dot{V}O_{2}\mathrm{peak}$)

Peak oxygen uptake ($\dot{V}O_{2}\text{peak}$) was determined using a grade exercise test on an electronically braked cycle ergometer (Lode Excalibur Sport; Lode B.V., Groningen, The Nether lands) in 10 of the participants. The test consisted of a 3‐min stage at 50 W followed by 25W increments every 1‐min to volitional exhaustion. Throughout the incremental exercise tests, metabolic and ventilatory data were continuously recorded via a gas exchange measurement system (True One 2400, Parvo Medics, Sandy, UT), which was calibrated before each testing session according to the manufacturer's instructions. $\dot{V}O_{2}\text{peak}$ was defined as the highest 15 sec value achieved during exercise. Peak O_2_ pulse was defined as the ratio of $\dot{V}O_{2}\text{peak}$ to peak heart rate. Due to testing equipment complications metabolic and ventilatory data could not be obtained in 1 participant. Maximal effort was confirmed by attainment of at least three criteria: (1) a respiratory exchange ratio > 1.1; (2) heart rate > 90% of age‐predicted maximum; (3) a plateau of $\dot{V}O_{2}$ defined as no expected increases (<150 mL min^−1^) in $\dot{V}O_{2}$ from the previous test stage; or (4) rating of perceived exertion > 17 on Borg's 6–20 scale. Heart rate was continuously derived from ECG (Q‐Stress ECG monitor, Quinton Instruments, Seattle, WA).

### Statistics

Paired *t* tests were used to compare normally distributed discrete variables before and after bed rest. Correlation analysis was performed using Pearson's method. Differences were considered statistically significant when *P* ≤ 0.05. To minimize the chances of a type II error due to a modest sample size, effect sizes (ES) were calculated as Cohen's d, which provides information on the magnitude of the difference between the groups. The threshold values for effect size were defined as small, moderate, and large effects as 0.2, 0.5, >0.8, respectively (Vincent and Weir 2012). Exploratory analysis of c‐miRNA expression was performed via Hierarchical clustering using an online R‐based tool ClustVis (Metsalu and Vilo 2015) that utilized principle component analysis and heatmap analysis. As part of an exploratory analysis the relative change in c‐miRNA with HDBR between men and women was evaluated by calculation of Cohen's D. All results are expressed as means ± standard deviation, unless indicated otherwise.

## Results

Following the bed rest period, body weight was significantly decreased (BDC‐1: 71.4 ± 8.7 kg, R + 0: 69.1 ± 7.9, *P* < 0.001), but no changes in total lean body mass (BDC‐3: 49.3 ± 10.5 kg, R + 0: 49.2 ± 10.3 kg, *P* = 0.85) or total fat mass (BDC‐3: 19.5 ± 5.4 kg, R + 0: 19.4 ± 5.2 kg, *P* = 0.91) were observed. Complete blood count revealed significant increases in red blood cell volume (BDC‐3: 4.8 ± 0.5 M·*μ*L^−1^, R + 0: 5.1 ± 0.5, *P* < 0.001), hemoglobin concentration (BDC‐3: 14.6 ± 1.3 g·dL^−1^, R + 0: 15.3 ± 1.3, *P* = 0.002), and hematocrit (BDC‐3: 42.1 ± 3.4%, R + 0: 43.6 ± 3.9, *P* = 0.01), but significant decreases in mean corpuscular volume (BDC‐3: 87.7 ± 2.7 fL,R + 0: 85.0 ± 3.0, *P* = 0.002) and mean corpuscular hemoglobin (BDC‐3: 30.5 ± 1.1 pg, R + 0: 29.9 ± 1.1, *P* = 0.003). Vitamin K1 (BDC‐3: 233 ± 72 ng·L^−1^, R + 0: 133 ± 61, *P* = 0.01) was significantly decreased following bed rest as was 1,25 Dihydroxyvitamin D (BDC‐3: 53 ± 17 pg·mL^−1^, R + 0: 33 ± 8, *P* = 0.003).

### Identification of differently expressed c‐miRNA in response to bed rest deconditioning

**Figure 1:**
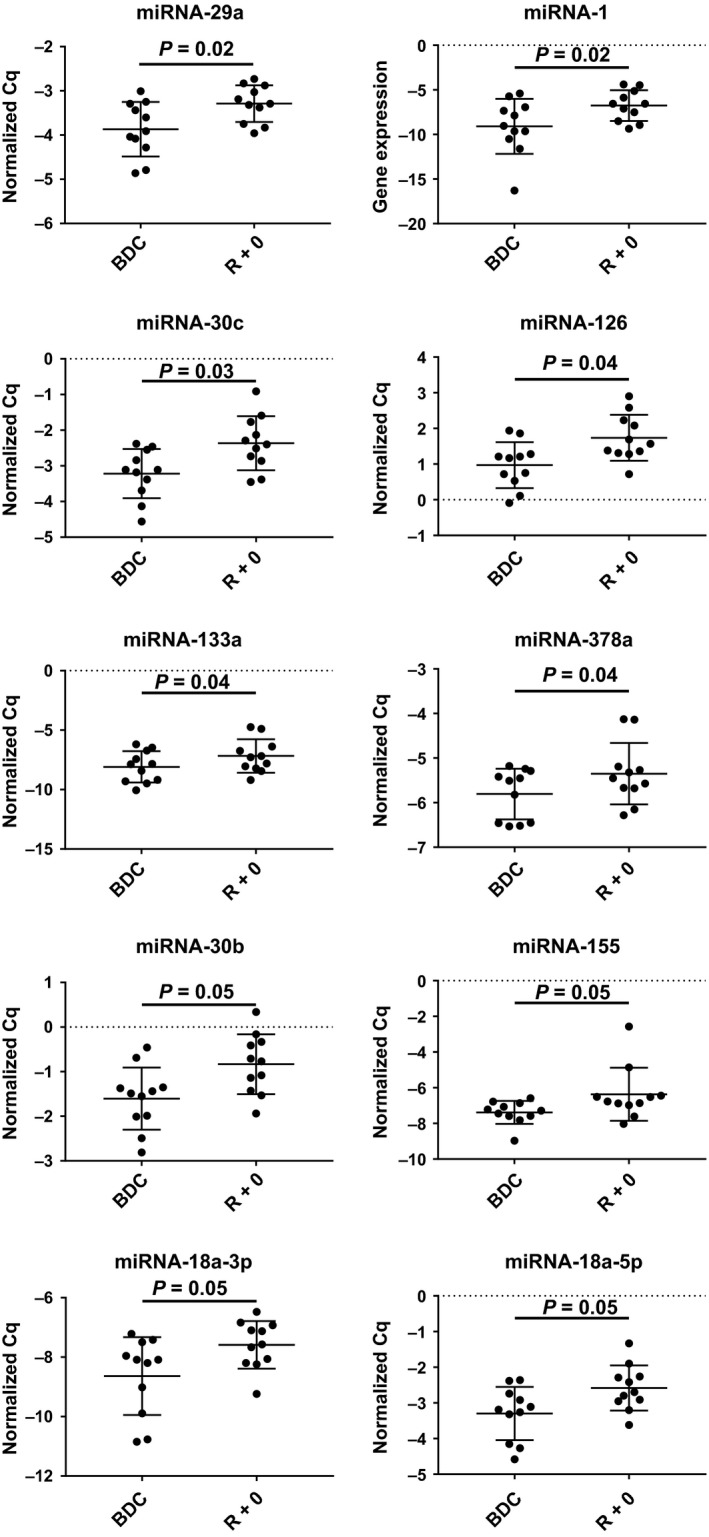
Expression levels of c‐miRNA in plasma at baseline (BDR) and after 30 days head‐down tilt bed rest (R + 0). Data are presented as mean ± SD.

**Figure 2:**
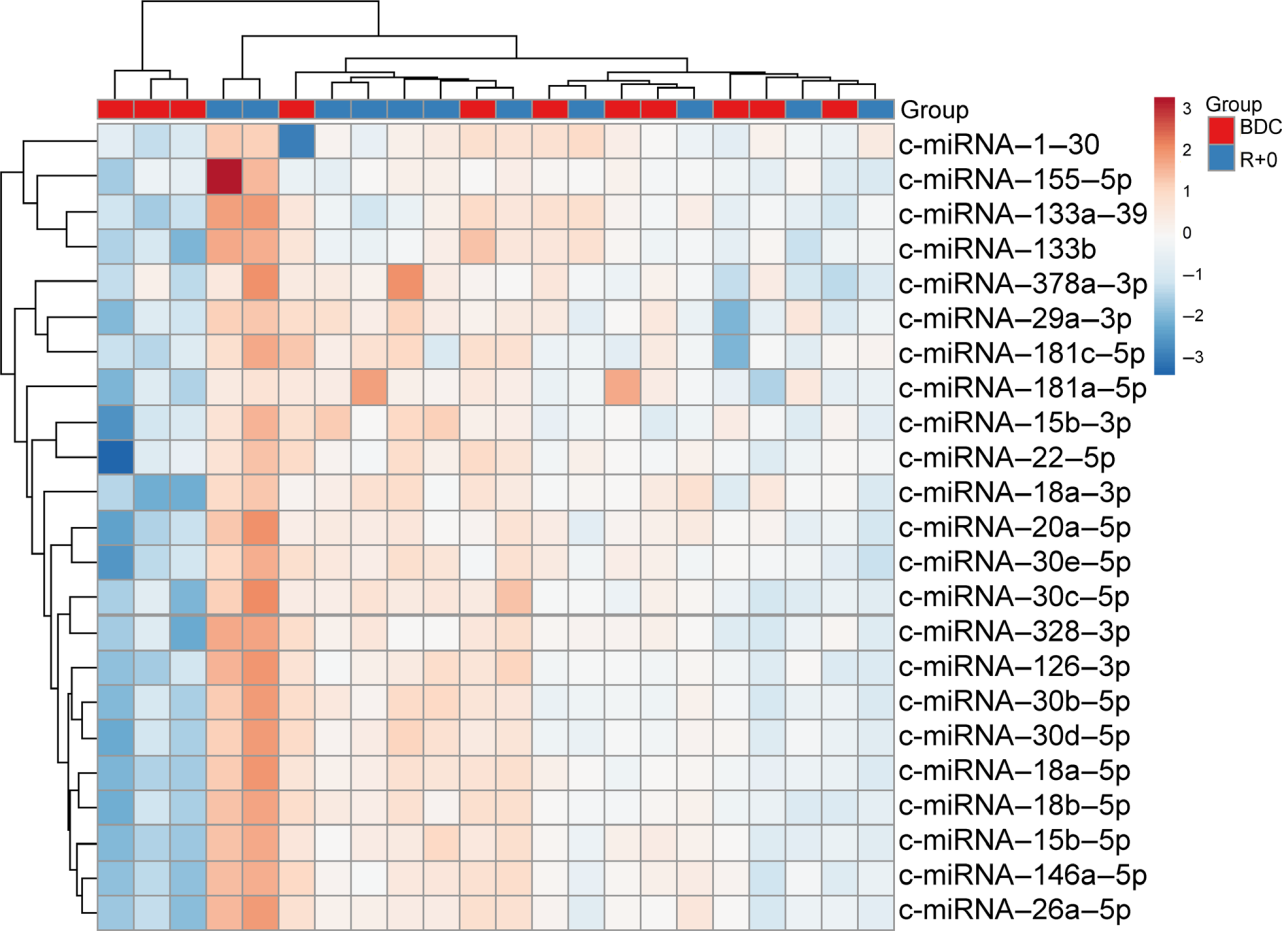
Heat map illustrating clusters of miRNA in response to 30‐day head‐down tilt bed rest.

**Figure 3:**
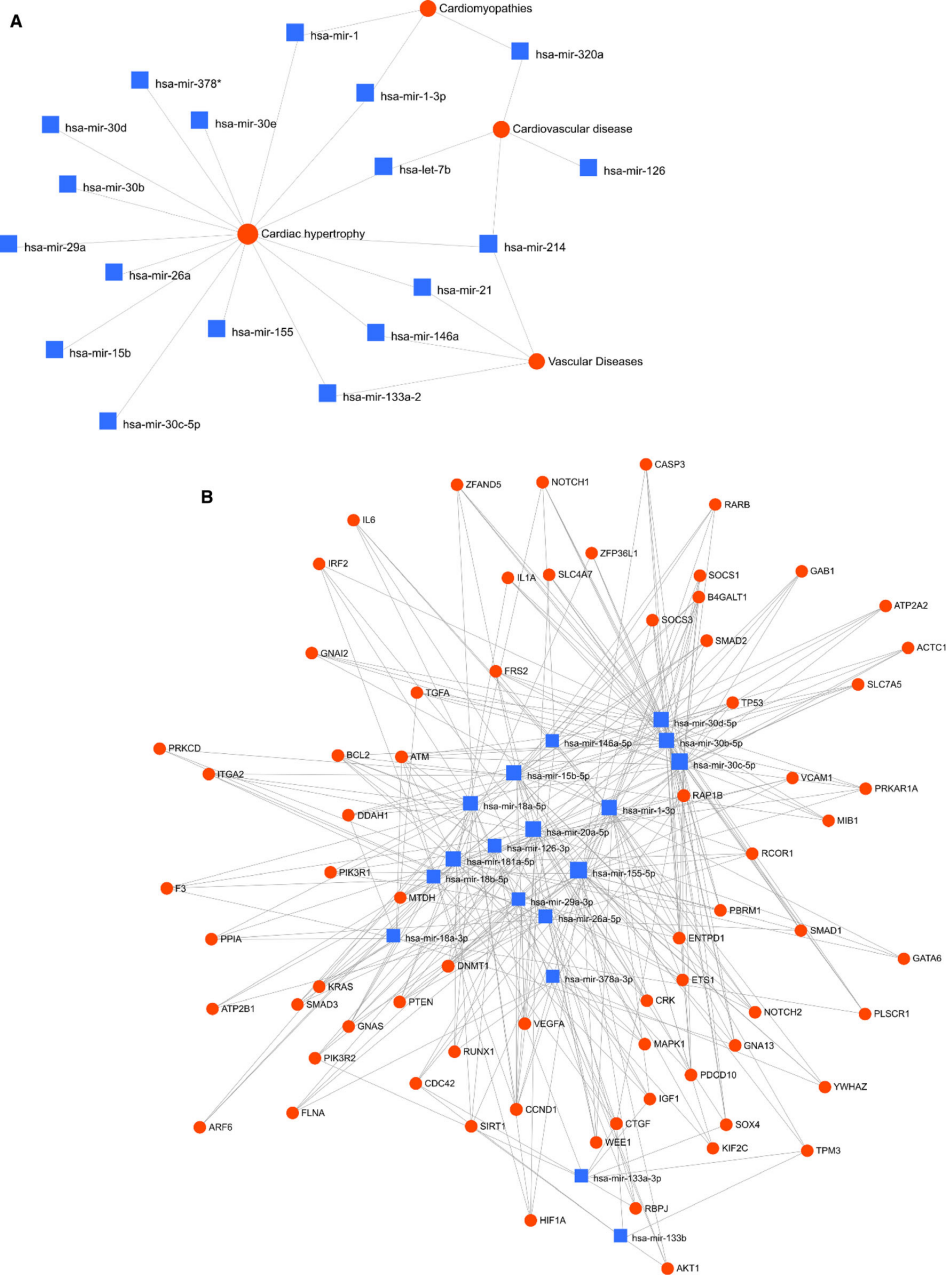
Functional enrichment analysis of c‐miRNA‐disease interactions (A) and c‐miRNA‐gene interactions (B) for the c‐miRNA significantly altered with bed rest.

Plasma c‐miRNA levels were assessed prior to and immediately following 30 days HDBR at a significant level of *P* ≤ 0.05 and ≥ 1.5‐fold change. Interestingly, 30 days bed rest had a pronounced effect on c‐miRNA expression. Quantification of circulating miRNA revealed that multiple cardiovascular‐related c‐miRNA were significantly upregulated following HDBR. Among the 45 c‐miRNA analyzed, 10 were found significantly increased in R + 0 plasma compared to BDC (Table 1 and Fig. 1). These were c‐miRNA‐29a, ‐1, ‐30c, ‐126, ‐133a, ‐378a, ‐30b, ‐155, and ‐18a. In addition, 16 c‐miRNA experienced a > 1.5‐fold increase with a moderate‐to‐high effect size, but were not statistically significant (Table 1). The differently expressed c‐miRNA were grouped into clusters according to their expression profile and are illustrated in Figure 2. Two major clusters were identified. Cluster A included c‐miRNA‐1, ‐155, ‐133a, and ‐133b, with Cluster B containing the remaining c‐miRNAs. Importantly, the c‐miRNAs included in Cluster A have been identified as regulators of cardiac function and hypertrophy (c‐miRNA‐133) (Care et al. 2007; Baggish et al. 2011; Mooren et al. 2014), atrial fibrillation and mitochondrial function (c‐miRNA‐1) (Zhang et al. 2014), skeletal muscle atrophy (c‐miRNA‐1) (Li et al. 2014; Rezen et al. 2014; Wang et al. 2017), and vascular control (c‐miRNA‐155) (Santovito et al. 2012; Sun et al. 2012). The second cluster contained multiple c‐miRNA that have been identified as regulators of cardiac hypertrophy (c‐miRNA‐15) (Tijsen et al. 2014), cardiac fibrosis (c‐miRNA‐22, ‐18) (Chen et al. 2013; Fang et al. 2015), mitochondrial function (c‐miRNA‐181)(Das et al. 2012), and aerobic exercise capacity (c‐miRNA‐20a) (Baggish et al. 2011; Mooren et al. 2014). As part of a secondary analysis a moderate effect size for changes in c‐miRNA‐1, ‐18a, and ‐15b between men and women were observed. Functional enrichment analysis revealed that several of the differentially regulated c‐miRNA had established relationships with key cardiovascular outcomes (Fig. 3A). Furthermore, GO and KEGG analysis revealed several c‐miRNA‐Gene interactions associated with cardiovascular processes including hemostasis, vascular development, angiogenesis, and cardiac development (Fig. 3B).

**Table 1:** Relative expression of circulating miRNAs

c‐miRNA	Mean ± SE	*P* value	Effect Size
hsa‐miRNA‐29a‐3p	1.7 ± 0.2	0.02	0.8
hsa‐miRNA‐1‐3p	82.3 ± 76.7	0.02	0.8
hsa‐miRNA‐30c‐5p	2.4 ± 0.6	0.03	0.8
hsa‐miRNA‐126‐3p	2.2 ± 0.4	0.04	0.7
hsa‐miRNA‐133a‐3p	2.7 ± 0.8	0.04	0.7
hsa‐miRNA‐378a‐3p	1.5 ± 0.2	0.05	0.7
hsa‐miRNA‐30b‐5p	2.2 ± 0.4	0.05	0.7
hsa‐miRNA‐155‐5p	3.7 ± 1.6	0.05	0.7
hsa‐miRNA‐18a‐3p	3.5 ± 1.3	0.05	0.7
hsa‐miRNA‐18a‐5p	2.1 ± 0.4	0.05	0.7
hsa‐miRNA‐30d‐5p	2.1 ± 0.4	0.06	0.6
hsa‐miRNA‐18b‐5p	2.1 ± 0.5	0.06	0.6
hsa‐miRNA‐15b‐5p	2.4 ± 0.6	0.07	0.6
hsa‐miRNA‐146a‐5p	2.3 ± 0.6	0.08	0.6
hsa‐miRNA‐26a‐5p	2.2 ± 0.5	0.09	0.6
hsa‐miRNA‐15b‐3p	1.8 ± 0.3	0.09	0.6
hsa‐miRNA‐133b	2.4 ± 0.7	0.09	0.6
hsa‐miRNA‐20a‐5p	1.8 ± 0.4	0.09	0.6
hsa‐miRNA‐328‐3p	2.2 ± 0.5	0.10	0.6
hsa‐miRNA‐499‐5p	2.6 ± 0.7	0.10	0.6
hsa‐miRNA‐30e‐5p	2.3 ± 0.7	0.11	0.5
hsa‐miRNA‐181c‐5p	2.8 ± 0.8	0.12	0.5
hsa‐miRNA‐181a‐5p	3.5 ± 1.0	0.13	0.5
hsa‐miRNA‐22‐5p	3.3 ± 1.7	0.14	0.5

Values are expressed as fold change relative to pre‐HDTBR (basal = 1.0).

### Cardiovascular morphologic and functional responses to bed rest deconditioning

**Figure 4:**
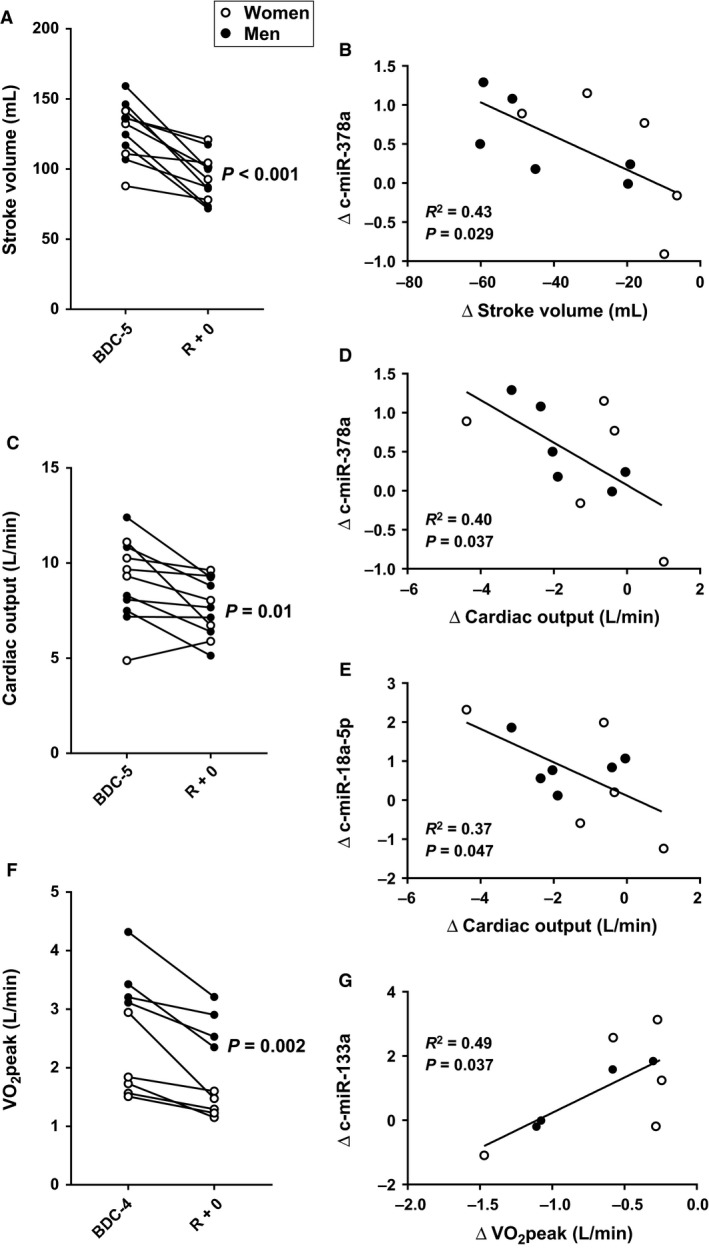
Alterations in c‐miRNA directly correlated with changes in cardiac output, stroke volume, and $\dot{V}O_{2}\text{peak}$. (A) Resting stroke volume measured prior to [baseline data collection (BDC)] and immediately upon completion of the bed rest protocol [recovery day 0 (R + 0)]. (B). Linear regression between changes in stoke volume and changes in miRNA‐378a. (C) Resting cardiac output measured prior and immediately upon completion of the bed rest protocol [recovery day 0 (R + 0)]. Linear regression between changes in cardiac output and changes in miRNA‐378a (D) and c‐miRNA‐18a (E). (F) $\dot{V}O_{2}\text{peak}$ significantly decreased with HDBR. (G) Linear regression between changes in $\dot{V}O_{2}\text{peak}$ and c‐miRNA‐133a.

Following 30 days head‐down tilt bedrest resting cardiac output and stroke volume were significantly decreased (Fig. 4), whereas resting heart rate was increased (71 ± 10 vs. 82 ± 12 bpm, *P* = 0.006).

Of the c‐miRNA that were differently expressed following bedrest miRNA‐378a and miRNA‐18a were significantly correlated with changes in cardiac output and stroke volume (Fig. 4). Head‐down tilt did not significantly change resting systolic arterial blood pressure (BDC‐1: 109 ± 7, R + 0: 113 ± 17, *P* = 0.22), but significantly increased diastolic (BDC‐1: 66 ± 4, R + 0: 75 ± 8, *P* < 0.001) and mean arterial blood pressure (BDC‐1: 80 ± 5, R + 0: 87 ± 8, *P* = 0.003). Resting heart rate was unchanged (BDC‐1: 62 ± 6, R + 0: 69 ± 11, *P* = 0.06).

Head‐down tilt bedrest resulted in a decrease in $\dot{V}O_{2}\text{peak}$ (BDC‐4: 2.63 ± 1.00 L·min^−1^, R + 0: 1.97 ± 0.79, *P* = 0.002) (BDC‐4: 36.4 ± 10.4 mL·kg^−1 ^min^−1^, R + 0: 27.9 ± 9.1, *P* = 0.001) that was significantly correlated with changes in miRNA‐133a (Fig. 4). Bed rest also resulted in significant decreases in peak power output (BDC‐4: 240 ± 67 W, R + 0: 193 ± 49, *P* < 0.001) and O_2_ pulse (BDC‐4: 14 ± 5 mL·beat^−1^, R + 0: 10 ± 4, *P* = 0.003), but not change in the peak heart rate response (BDC‐4: 183 ± 14 beat·min^−1^, R + 0: 186 ± 11, *P* = 0.13).

## Discussion

The purpose of this investigation was to evaluate the differential expression of cardiovascular‐health related c‐miRNA following 30 days HDBR and to determine how they relate to changes in key parameters of cardiovascular function. As such, there are several important findings that support further exploration of miRNAs as regulators of the cardiovascular responses to bed rest. First, multiple c‐miRNA with cardiac relevance were upregulated following bed rest. Second, differentially expressed c‐miRNA, were categorized based on their expression profile, with several c‐miRNA‐Gene interactions associated with cardiovascular processes. Third, changes in c‐miRNA‐378a and ‐18a correlated with changes in cardiac output and stroke volume, established major consequences of prolonged bed rest. Finally, changes in c‐miRNA‐133a was significantly correlated with changes in aerobic capacity. These observations provide novel insight into the potential regulatory role of miRNA in the myriad cardiovascular responses to HDBR and their potential role as biomarkers of important cardiovascular parameters.

Although several cardiovascular changes are well defined after microgravity, to our knowledge this study is the first to demonstrate an up‐regulation of several cardiovascular‐health related c‐miRNA during 30 days simulated microgravity via HDBR. To date, unique profiles of c‐miRNA expression have been identified for both physiological and pathological cardiovascular remodeling, and therefore may provide insight into the regulation of the cardiovascular responses to prolonged microgravity exposure. Of the 45 c‐miRNA investigated, miRNA‐29a, ‐1, ‐30c, ‐126, ‐133a, ‐378a, ‐30b, ‐155, and ‐18a were all upregulated following HDBR. Relevant to our findings, miRNA‐29a has been shown to be a regulator of fibrosis in cardiac tissue. Roncarati et al. (2014) demonstrated a significant positive relationship between c‐miRNA‐29a and myocardial fibrosis in patients with hypertrophic cardiomyopathy. While, miRNA‐29 appears to prevent excess collagen in the extracellular matrix following myocardial infarction via cardiac fibroblasts (van Rooij et al. 2008), targeted deletion of miRNA‐29 expression in the cardiac myocyte has been shown to prevent fibrosis in a mouse model of pressure overload cardiac hypertrophy (Sassi et al. 2017). Interestingly, miRNA‐29 expression has also been shown to be altered in a rodent model of cardiac atrophy (El‐Armouche et al. 2010). El‐Armouche et al. (2010) subjected rats to decreases in cardiac workload and subsequent cardiac atrophy via heterotopic heart transplantation and increases in cardiac workload via aortic stenosis. Importantly, in the left ventricle tissue miRNA‐19a was upregulated with cardiac atrophy and unchanged with aortic stenosis‐induced hypertrophy (El‐Armouche et al. 2010). Similar to miRNA‐29, the miRNA‐17‐92 cluster, which encodes six miRNAs (miRNA‐17, miRNA‐18a, miRNA‐19a, miRNA‐19b, miRNA‐20a, and miRNA‐92a‐1), has been implicated in the regulation of cardiomyocyte proliferation (Chen et al. 2013) and myocardial fibrosis (van Almen et al. 2011). Importantly, miRNA‐18a has been shown to target connective tissue growth factor and thrombospondin‐1 in the context of myocardial fibrosis (Chen et al. 2013). As such, miRNA‐18a has been shown to be significantly up‐regulated in patients with diffuse fibrosis and was significantly correlated with degree of left ventricular fibrosis (Fang et al. 2015). Following simulated microgravity, cardiac performance is decreased in part to decreases in left ventricular dynamic stiffness suggesting that the heart undergoes some physiologic remodeling (Levine et al. 1997). In this study, miRNA‐18a was significantly correlated with cardiac output, which lends to the speculation that miRNA‐18a regulation of cardiac interstitial remodeling may be involved. Importantly, miRNA‐18a, like many c‐miRNAs, is also involved in peripheral vascular responses. Expression of miRNA‐18a has been shown to contribute to vascular smooth muscle differentiation following carotid artery injury (Kee et al. 2014).

As revealed by functional enrichment analysis, several of the upregulated miRNA have been linked to the regulation of cardiac hypertrophy. However, El‐Armouche et al. (2010) recently demonstrated divergent miRNA expression in mechanical load‐induced cardiac hypertrophy and atrophy, therefore the miRNA expression profiles regulating cardiac mass in pathological conditions may not translate to bed rest. miRNA‐378 is expressed in the mammalian heart and has recently been shown to predict left ventricular hypertrophy in patients with aortic stenosis. Chen et al. (2014) demonstrated that c‐miRNA‐378 were significantly lower in aortic stenosis patients with left ventricular stenosis compared to nonhypertrophied patients and health controls. Furthermore, they demonstrated a significant correlation between left ventricular mass and c‐miRNA‐378. This work is further supported by in vivo and in vitro work demonstrating a role for miRNA‐378 in mediating cardiomyocyte hypertrophy. Ganesan et al. (2013) demonstrated in vitro a significant attenuation in neonatal rat cardiomyocyte hypertrophy following transfection with miRNA‐378 that was consequently reversed with use of anti‐miRNA‐378. The authors also demonstrated in vivo a significant decrease in miRNA‐378 expression of 54% in a rodent model of chronic pressure overload induced hypertrophy. Furthermore, when these pressure overloaded rats were transfected with miRNA‐378 partial prevention of left ventricular hypertrophy was achieved, providing evidence for an antihypertrophic activity of miRNA‐378 in the heart (Ganesan et al. 2013). Interestingly, in our small cohort, we observed a similar relationship between stroke volume and cardiac output and c‐miRNA378, suggesting a partial regulatory role of miRNA‐378 in mediating cardiac performance presumably through transcriptomic control of cardiac mass.

To date, only a few have focused on the relationship between aerobic exercise capacity, which is an integrative measurement of total cardiovascular capacity (Ade et al. 2015, 2017), and c‐miRNA expression. Recent work by Baggish et al. (2011) and Mooren et al. (2014) has demonstrated a potential role of c‐miRNAs as mediators of exercise‐induced cardiovascular adaptation. Following 90 days aerobic exercise training, significant increases in several c‐miRNA, with c‐miRNA‐146a and ‐20a being significantly correlated with training induced increases in $\dot{V}O_{2}\text{peak}$. In this study, no significant increases in these c‐miRNAs occurred suggesting a differential regulation of these miRNA with respect to training versus detraining. Interestingly, however, Baggish et al. (2011) demonstrated in a group of athletes that $\dot{V}O_{2}\text{peak}$ was significantly correlated with the expression of c‐miRNA‐133a. This finding is consistent with this study's findings that changes in c‐miRNA‐133a expression were related to the change in bed rest‐induced reductions in $\dot{V}O_{2}\text{peak}$. Recent work in animal models of aerobic exercise training and cardiac hypertrophy have provided some insight into the potential role of miRNA‐133a in cardiovascular adaptation. Soci et al. (2011) demonstrated a significant decrease in miRNA‐133a expression in left ventricular tissue of rodents following several weeks of swim training, which occurred concomitantly with increases in $\dot{V}O_{2}\text{peak}$, left ventricular hypertrophy, and citrate synthase activity. This potential role for miRNA‐133a in the hypertrophic response, and potential exercise capacity, is further supported by the work of Care et al. (2007). These authors not only confirmed a decreased left ventricular miRNA‐133a expression with exercise training in rodents, but also demonstrated in vitro and in vivo that decreasing miRNA‐133a expression increased hypertrophic parameters (Care et al. 2007). Collectively, the previous work coupled with our results highlight how simulated microgravity, via head‐down tilt bed rest, alters important and specific c‐miRNAs described as responsible for controlling various aspects of cardiac remodeling and aerobic exercise capacity.

In this study, several c‐miRNA obtained at the end of the 30 day HDBR were correlated with changes in key cardiovascular parameters. This suggests that certain miRNAs may contribute to an effect on the well‐known variability in physiological responses to HDBR. Several HDBR studies report considerable person‐to‐person variability in cardiac and aerobic exercise capacity responses. Following Skylab and shuttle missions, the change in left ventricular volumes and mass are variable between crewmembers. Perhonen et al. (2001) reported that following a 10 day shuttle mission (STS‐55), left ventricular mass was only decreased in 3 of the 4 crewmembers with one having a near 10 g increase post flight. The same group reported an average mean decrease in left ventricular mass of 8% following 6 weeks of bed rest, but a standard deviation of > 2%, suggesting a considerable variability in the response. This variability in mass was mirrored by a variable change in left ventricular end‐diastolic volume and continued to exist after 12 weeks. In addition to changes within the left ventricle, variable decreases in aerobic exercise capacity following bed rest and spaceflight have been reported (Ade et al. 2015). While changes in plasma volume are likely contributing to some of the variability in these cardiac responses, the paralleled decrease in cardiac mass is suggestive of some cardiac remodeling that may be partly mediated by miRNA regulatory signals. Furthermore, long‐duration flights appear to results in multiple cardiovascular adaptations, with several directly mediating the postflight decreases in aerobic exercise capacity. The findings from this study, while speculative, suggest a role for transcriptomic signaling via miRNA in mediating these responses.

While the correlation between our post‐HDBR c‐miRNA in mediating physiological changes is insightful, the lack of information on the temporal responses of c‐miRNA expression and cardiac adaptation in this study must be considered as a limitation. Westby et al. (2016) demonstrated a linear decrease in left ventricular end‐diastolic volume over the course of a 60 day HDBR period. A similar response was observed when these authors reviewed the temporal decrease in left ventricular wall mass over an 84 day HDBR period, highlighting the progressive changes in cardiac structure that occur in response to simulated microgravity (Westby et al. 2016). We and others have also demonstrated that aerobic exercise capacity decreases in a linear fashion as a function of HDBR duration over the first 30 days (Ade et al. 2015). It is, however, unknown if the expression of our described c‐miRNAs were altered in a similar manner over the 30 day HDBR period. Furthermore, the temporal c‐miRNA response observed in the circulation may be delayed compared to the miRNA responses in the tissue from which they originated. Therefore, we cannot be certain if the differentially expressed c‐miRNA observed in this study were upregulated at the beginning or progressed over time, making interpretation of their direct role in mediating the observed cardiovascular changes limited. However, the significant findings of this study clearly demonstrate an altered regulation of c‐miRNA induced by HDBR that will required additional work to both evaluate the temporal changes in tissue and the circulation and use techniques in animal models to target the identified miRNA from this study with miRNA mimics and anti‐miRNAs.

Although, this study demonstrates significant changes in c‐miRNA expression that is associated with cardiovascular perturbations resulting from HDBR there are some experimental considerations to be noted. First, this study utilized a modest sample size consisting of men and women. This sample was based in part on the available research space at the :envihab bed rest facility and the cost of performing a head‐down tilt bed rest study. Second, a nonbed rest control group was not included in this study in accordance with multiple previous bed rest studies (Levine et al. 1997). Another limitation of this study was the obtainment of only pre‐ and post‐bed rest measurements, instead of interval measurements. This would have allowed for the evaluation of the time course of the physiological responses associated with HDBR to be observed. Future work will be required to evaluate the relationship between the time course changes in c‐miRNA and key physiological outcomes. Lastly, our group of men and women were relatively untrained based on their pre‐rest $\dot{V}O_{2}\text{peak}$. Although significant decreases in stroke volume, cardiac output, and aerobic exercise capacity were observed, it is likely an even greater magnitude of change would have been observed in more fit participants. Therefore, the magnitude of the responses observed in this study may not be representative of what would occur inflight. This is also an important consideration given that exercise training impact the c‐miRNA profile and that inflight astronauts perform regular exercise training during long‐duration flights.

In conclusion, this study found significant alterations in cardiovascular‐health related c‐miRNAs following 30 days sedentary HDBR. Importantly, several of these c‐miRNAs were significantly correlated with changes in stroke volume, cardiac output, and maximal aerobic exercise capacity. We speculate that miRNA may play an effect on modulating the cardiovascular responses associated with prolonged microgravity exposure. Future work will need to confirm these results in a larger bed rest cohort and in the true spaceflight environment.

## Conflict of Interests

None declared.
